# The clinical efficacy of laser in the nonsurgical treatment of peri-implantitis: a systematic review and meta-analysis

**DOI:** 10.1186/s40729-024-00570-x

**Published:** 2024-11-14

**Authors:** Nengwen Huang, Yang Li, Wen Li, Rui Zhao, Yanjing Ou, Jiang Chen, Jinjin Li

**Affiliations:** 1 Department of Stomatology, The First Affiliated Hospital of Zhengzhou University, No.1 Jianshe Dong Road, ErQi District, Zhengzhou, Henan China; 2 Fujian Key Laboratory of Oral Diseases, School and Hospital of Stomatology, Fujian Medical University, 46 Yangqiao Zhong Lu, Gulou District, Fuzhou, Fujian China; 3 Department of Oral and Maxillofacial Surgery, The First Affiliated Hospital of Zhengzhou University, Zhengzhou, Henan China

**Keywords:** Peri-implantitis, Nonsurgical treatment, Laser, Systematic review

## Abstract

**Objective:**

To systematically assess studies regarding the efficacy of lasers in the nonsurgical treatment of peri-implantitis.

**Methods:**

Electronic and manual searches were performed by two reviewers independently. Randomized controlled trials (RCTs) comparing lasers vs. mechanical debridement or air abrasive on primary outcome (probing depth (PD)) and secondary outcomes (bone loss, bleeding on probing (BOP), clinical attachment level (CAL) and plaque index (PI)) were included. Data extraction and quality assessment were conducted independently. Weighted mean difference (WMD) or standardized mean difference (SMD) and 95% confidence interval (CI) were calculated for continuous outcomes. Publication bias, leave-one-out analysis and GRADE assessment were conducted.

**Result:**

13 eligible publications were included in the review and 12 in the meta-analysis. Solid-state lasers significantly improved in PD (WMD = -0.39, 95% CI (-0.70, -0.09), *p* = 0.01, moderate-certainty evidence), BOP (SMD =-0.76, 95% CI (-1.23, -0.28), *p* = 0.002, moderate-certainty evidence) and CAL (WMD =-0.19, 95% CI (-0.39, -0.00), *p* = 0.05, moderate-certainty evidence), but not in bone loss (WMD = 0.03, 95% CI (-0.13, 0.18), *p* = 0.74, low-certainty evidence) and PI (SMD =-0.19, 95% CI (-0.42, 0.04), *p* = 0.11, moderate-certainty evidence) compared with the control group. However, the diode lasers showed no clinical advantages. No publication bias was detected, and leave-one-out analysis confirmed the robustness of findings.

**Conclusion:**

In the nonsurgical treatment of peri-implantitis, solid-state lasers yielded positive influence in term of PD, BOP and CAL, while diode laser provided no beneficial effect. Future well-designed large RCTs are still needed, considering the limitations of included studies.

**Clinical relevance:**

This review aimed to guide clinicians in choosing the appropriate laser for peri-implantitis, enhancing treatment strategies and attaining better outcomes.

**Supplementary Information:**

The online version contains supplementary material available at 10.1186/s40729-024-00570-x.

## Introduction

Peri-implantitis is a plaque-associated disease occurring around osseointegrated implants, involving soft and hard tissues. It features symptoms like bleeding, progressive loss of supporting bone, and other symptoms, eventually resulting in implant failure [1]. In many ways, peri-implantitis resembles periodontitis; but in contrast to natural teeth, the coarseness and structures of the implant surfaces render the treatment more challenging [2]. Moreover, the progression of peri-implantitis appears to be more rapid than what is observed in periodontitis. Retrospective analysis confirmed that the bone loss pattern in peri-implantitis was nonlinear, with an accelerating progression and increasing variability over time [3]. However, due to the variation of diagnostic criteria for peri-implantitis, it is challenging to ascertain its global prevalence rate [4]. Recent available studies have shown a diverse range of prevalence for peri-implantitis (8.9–45% at the patient level, ranging from 4.8 to 23.0% at the implant level) [5]. Consequently, the escalating pattern and widespread nature of this condition undeniably categorize it as a significant oral health issue, affecting millions and exacerbating the burden on patients.

Since the etiology of peri-implantitis is intimately related to bacterial biofilms, the majority of suggested treatments emphasize altering biocompatibility and minimizing bacterial plaque. Due to the screw threads or rough surfaces of implants, along with the specific morphology of the implant neck, which predispose to bacterial adhesion and biofilm development, achieving effective therapeutic outcomes through mechanical debridement (MD) alone is notably challenging. Hence, in addition to MD, a variety of supplementary strategies, encompassing non-surgical interventions and surgical treatments, along with multifaceted therapies for surface decontamination, have been suggested [6]. However, the open trauma from surgical treatment may cause the permanent retreat of periodontal soft tissue impacting aesthetics. With the popularization of non-invasive and minimally invasive concepts, non-surgical treatments like medication, photodynamic therapy (PDT) and laser therapy, have been gradually applied in clinics [7, 8].

Laser applications in the treatment of periodontal and implant diseases have seen a gradual expansion, driven by a growing body of research utilizing various types of lasers, including gas laser (CO_2_), semiconductor laser (diode) and solid-state laser (erbium-doped: yttrium aluminum garnet, Er: YAG; neodymium-doped: yttrium aluminum garnet, Nd: YAG; erbium, chromium-doped: yttrium, scandium, gallium, garnet; Er, Cr: YSGG) [9]. Employed with exacting parameters, lasers provide a comprehensive and nuanced approach to implant care. They can effectively eliminate plaque buildup on the implant surface, enhance surface texture to facilitate osseointegration, and gently eliminate hyperplastic or infected gum tissue—all achieved through non-invasive means.

Clinical trials evaluating the effectiveness of nonsurgical laser therapy for peri-implantitis have yielded mixed results. Kang et al. and Alpaslan et al. have indicated that the utilization of Er, Cr: YSGG laser in the non-surgical management of peri-implantitis offers enhanced benefits when compared to the sole reliance on conventional mechanical debridement [10, 11]. However, Renvert et al. reported that Er: YAG offers marginal advantages when used for nonsurgical treatment of peri-implantitis [12]. Furthermore, recent studies have shown that CO_2_ and diode lasers are not effective in efficiently removing plaque from titanium implants [13, 14].

Due to the limited number and types of studies included in the above report, additional evidence is imperative to evaluate the clinical value of laser therapy. Consequently, the aim of this systematic review and meta-analysis is to comprehensively assess the clinical efficacy of laser therapy in the non-surgical management of peri-implantitis.

## Methods

### Patient and public involvement

No patients were involved in the study.

### Study design

This systematic review was reported in accordance with recommendations of the Preferred Reporting Items for Systematic Reviews and Meta-Analyses (PRISMA) statement and registered in the PROSPERO database (CRD42023425210) [15].

### Focused question

The present systematic review addressed the following focused question that was structured according to the PICO format: “What is the clinical efficacy of laser therapy in the non-surgical treatment of peri-implantitis?” Population: the adult patients were diagnosed with peri-implantitis based on case definition used in the publications. Intervention: any type of laser therapy to the non-surgical implant surface debridement. Comparison: nonsurgical implant surface debridement or air-abrasive. Outcomes: The primary outcome was the alteration in probing depth (PD). Secondary outcomes encompassed changes in bone loss, clinical attachment level (CAL), bleeding on probing (BOP), and plaque index (PI).

### Search strategy

A critical electronic search was conducted in the following databases, mainly including PubMed, Scopus, Cochrane Central Register of Controlled Trials and Web of Science, covering the period from January 1, 2000, to June 18, 2024. In addition, the gray literature was searched using the database System for Information on Gray literature in Europe (http://www.opengrey.eu). Several journals on implantology followed were also manually retrieved: Journal of Periodontology; Clinical Oral Implants Research; Journal of Clinical Periodontology; International Journal of Oral and Maxillofacial Implants; Clinical Implant Dentistry and Related Research. When needed, we would contact with the corresponding authors for missing data or relevant information. Primary screening and assessment of potential articles was performed independently by two reviewers. Any disagreement during first stage of screening was resolved by discussion or consulting a third reviewer.

Keywords from the Medical Subject Headings (MeSH) identified by an asterisk symbol (*) and free text terms were the following: laser therapy* or laser* or laser therapy or laser and peri-implantitis* or peri-implant infections or peri-implantitis or peri-implant bone loss or peri-implant defect or peri-implant bone loss. The detailed search strategy for each database was shown in supplemental file 1. Endnote X7 was used for electronic title management. Only English language articles were considered.

### Study inclusion and exclusion criteria

During the first stage of the study selection, studies were considered eligible for meeting the following inclusion criteria: (1) study types: randomized controlled clinical trial (RCT, parallel group design) or randomized controlled cross-over studies in humans; (2) comparison of laser therapy vs. mechanical debridement or air-abrasive; (3) clinical data changes of peri-implantitis (i.e., PD, CAL, BOP, PI) or bone level before and after treatment. (4) a follow-up assessment no less than 6 months. At the second stage of the selection, eligible studies acquired in the first stage were identified according to the following exclusion criteria: (1) in vitro and animal studies, letter to the editor, review articles, interviews, opinion article, monographs and meta-analysis; (2) unclear peri-implantitis identification; (3) studies with no completed or unavailable data obtained even by contacting the authors; (4) patients who received surgical treatments; (5) inclusion of less than five patients.

### Risk of bias (quality) assessment

The included studies underwent a quality assessment with the Revised Cochrane risk of bias tool for randomized trials (RoB2) [16]. Briefly, five domain areas (randomization, allocation concealment, participants and professionals blinded to the study, blinding of outcome assessment, and other bias) were evaluated. The overall bias was classified as “high risk of bias” (high), “low risk of bias” (low), or “unclear risk of bias” (?). After screening the articles, two reviewers conducted the assessment independently. Any disagreement was resolved by discussion or consulting a third reviewer.

### Certainty assessment

The Grading of Recommendations, Assessment, Development, and Evaluation (GRADE) tool (available at https://gdt.gradepro.org/app/#projects, accessed on 5 October 2024) was used to evaluate the certainty of evidence by two authors independently. This tool assesses the study design and takes into account factors like the risk of bias, imprecision, inconsistency, indirectness of evidence, and publication bias, which may be used to determine the quality of evidence. Each factor is judged as “no serious”, “serious”, or “very serious”, enabling the classification of evidence quality into “high, moderate, low, or very low”.

### Data extraction and data item

Data and information from the included articles were retrieved and collected into predesigned table by two reviewers independently: (1) study identification: author’s name, the year of publication; (2) study type (RCTs), (3) population: numbers of patients, numbers of implants, age range, smoking habits; (4) definition of peri-implantitis; (5) laser parameters; (6) type of treatment; (7) clinical outcomes; (8) follow-up. Any discrepancies were resolved by discussion with a third reviewer.

Concerning clinical parameters, the changes of PD were defined as the primary outcomes. The secondary outcomes in this review included the changes of BOP, PI, CAL and bone loss.

### Statistical analysis

Statistical heterogeneity between studies was assessed by Q and I^2^ test. When a p value of Q statistic was < 0.1, it was defined as an indicator of heterogeneity. The threshold for the interpretation of I^2^ values was also used to estimate the heterogeneity as follows: 0–30% low heterogeneity, 30–60% moderate heterogeneity, and > 60% substantial heterogeneity. Subgroup analysis should be conducted to analyze the sources of heterogeneity, if I^2^ > 50% or *p* < 0.10 [17, 18]. Differences between experimental and control group were expressed as the weighted mean difference (WMD) with 95% confidence interval (CI) for PD, bone loss and CAL; and the standardized mean difference (SMD) with 95% CI for BOP and PI, using the random effect models. As regards continuous outcomes, the mean difference and standard deviation had to be collected. If data were not reported as mean difference, the mean difference was calculated and the standard deviation was estimated using the formula: r_d_ = sqrt (r_1_^2^/n_1_ + r_2_^2^/n_2_). Publication bias was performed objectively by Egger’s tests, in which a p value < 0.05 indicating publication bias [19]. Sensitivity analysis (leave-one-out analysis) was performed to assess the stability of results by sequential omission of included studies [20]. The meta-analysis was performed using the Review Manager 5.3 and Stata 17.

## Result

### Study selection

**Fig. 1:**
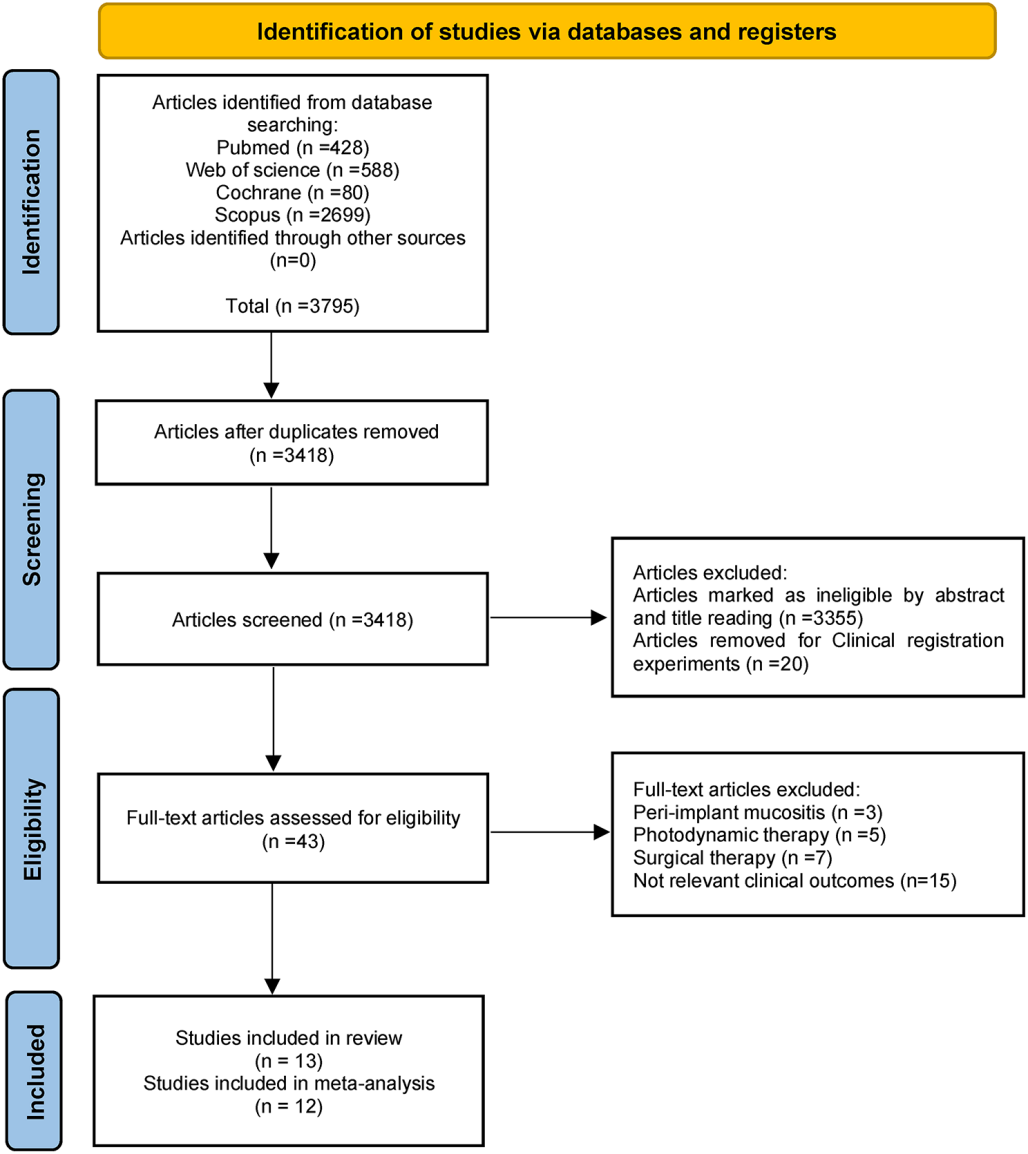
Flow diagram of literature search strategy and inclusion and exclusion criteria

A total 3795 articles were potentially identified through electronic and manual search. After removing the duplicates, 43 articles were included by screening the titles and abstracts. These studies were evaluated by reading the full texts and 13 articles were included into the review, but among them, one article was excluded from meta-analysis because of unavailable data (Fig. 1) [10, 11, 21–31].

### Study characteristics

The main characteristics of the included studies were shown in Table 1. All the studies were RCTs and published in English up to 06/2024. Smokers were excluded except for these three studies [25, 26, 29]. Oral hygiene instruction (OHI) was performed in all studies except two studies [21, 30]. The number of patients in included studies ranged from 10 to 100, and the number of implants ranged from 25 to 100. The included studies involved most types of lasers, including Nd: YAG, Er: YAG, Er, Cr: YSGG and diode laser. Although there are slight differences about the definition of peri-implantitis among included studies, they are broadly consistent with the 2017 classification [1]. For the convenience of the reader, the definitions used in each study to diagnose peri-implantitis were listed in Table 1.

**Table 1:** Characteristics of the included studies

Study	Type	Population	Case definition	Laser parameters	Treatment	Mean (SD) outcome	Follow- up
Persson [26]	RCT Parallel	42 patients, 100 implants Test: 21 patients, 55 implants; age: 68.5 ± 6.4 years Control: 21 patients; 45 implants; age: 68.9 ± 12.5 years	RBL ≥ 2 mm; PD ≥ 5 mm with BOP and/or pus	Er: YAG laser at an energy level of 100 mJ/pulse and 10 Hz (12.7 J/cm^2^)	Er: YAG laser + OHI (test) air-abrasive + OHI (control)	PD (mm): -0.9 ± 0.8 (test) -0.8 ± 0.5 (control)	6 M
Renvert [25]	RCT Parallel	42 patients, 100 implants Test: 21 patients, 55 implants; age: 68.5 ± 6.4 years Control: 21 patients, 45 implants; age: 68.9 ± 12.5 years	RBL > 3 mm; PD ≥ 5 mm with BOP and/or pus	Er: YAG laser: energy level of 100 mJ/pulse and 10 Hz (12.7 J/cm^2^)	Er: YAG laser + OHI (test) air-abrasive + OHI (control)	PD (mm): -0.8 ± 0.5 (test) -0.9 ± 0.8 (control) RBL (mm): -0.3 ± 0.9 (test) -0.1 ± 0.8 (control)	6 M
Schwarz [24]	RCT Parallel	20 patients, 32 implants Test: 10 patients, 16 implants; mean age: 48 years Control: 10 patients, 16 implants; mean age: 51 years Smokers excluded	PD ≥ 4 mm; RBL; BOP and/or suppuration	Er: YAG laser: 2.94 μm wavelength, 100 mJ/pulse (12.7 J/cm^2^), 10 pps	Er: YAG + OHI (test) MD + OHI + pocket irrigation with 0.2% CHX and subgingival application of 0.2% CHX gel (control)	CAL (mm): -0.7 ± 0.9 (test) -0.6 ± 1.45 (control) BOP (%): -52 ± 14.48 (test) -21.57 ± 17.83 (test) PD (mm): -0.8 ± 1.15 (test) -0.7 ± 1.45 (control) GR (mm): 0.1 ± 0.6 (test) 0.1 ± 0.8 (control) PI: 0 ± 0.46 (test) 0 ± 0.5 (control)	6 M
Strauss [22]	RCT Parallel	19 patients, 34 implants Test: 19 implants Control: 15 implants Age: ≥ 18 years Smokers excluded	PD > 4 mm with suppuration and/or BOP, RBL	Nd: YAG laser: 3.6 watts at 20mHz, 4 J/mm probing depth; 100 µs pulse	Nd: YAG laser + MD + CHX irrigation + occlusal adjustment + OHI (test) MD + CHX irrigation + occlusal adjustment + OHI (control)	PD (mm): -1.89 ± 1.33 (test) -1.36 ± 2.01 (control) RBL (mm): -0.41 ± 0.92 (test) -0.26 ± 0.64 (control)	12 M
Schwarz [23]	RCT Parallel	18 patients, 36 implants Test: 10 patients, 20 implants; age: 56 ± 14 years Control: 8 patients, 16 implants; age: 52 ± 11 years Smokers excluded	PD: 4–6 mm (moderated) > 7 mm (advanced); BOP/purulence	Er: YAG laser: 2.94 μm wavelength, energy level of 100 mJ/pulse (12.7 J/cm^2^), 10 Hz	Er: YAG + OHI (test) MD + OHI + pocket irrigation with 0.2% CHX and subgingival application of 0.2% CHX gel (control)	**6 months**: CAL (mm): moderated − 0.52 ± 0.34 (test) -0.23 ± 0.46 (control) advanced: -0.37 ± 0.57 (test) -0.33 ± 0.82 (control) PD (mm): moderated − 0.78 ± 0.21 (test) -0.32 ± 0.41 (control) advanced: -0.68 ± 0.39 (test) -0.48 ± 0.85 (control) BOP (%) moderated: -67 ± 25 (test) -42 ± 19 (control) PI moderated: -0.2 ± 0.7 (test) -0.25 ± 0.75 (control) **12 months**: CAL (mm): moderated − 0.23 ± 0.11 (test) -0.05 ± 0.46 (control) advanced − 0.18 ± 0.58 (test) -0.23 ± 0.81 (control) PD (mm): moderated − 0.5 ± 0.28 (test) -0.15 ± 0.41 (control) advanced − 0.49 ± 0.4 (test) -0.39 ± 0.85 (control) BOP (%): moderated -59 ± 22 (test) -32 ± 17 (control) PI: moderated − 0.3 ± 1 (test) -0.35 ± 1.05 (control)	6,12 M
Chen [21]	RCT Parallel	23 patients, 25 implants Test: 11 patients, 13 implants; Control: 12 patients, 12 implants Smokers excluded	RBL and/or PD < 7 mm with BOP	Er: YAG laser: 2940 nm wavelength, 100 mJ/pulse, 10 Hz frequency	Er: YAG laser (test) MD (control)	PD (mm): -0.85 ± 1.95 (test) -0.42 ± 1.72 (control) RBL (mm): -0.12 ± 2.66 (test) 0.19 ± 1.78 (control) BOP (sites): -0.85 ± 2.18 (test) -0.84 ± 1.82 (control)	6 M
Kang [11]	RCT Parallel	23 patients, 64 implants Test: 13 patients, 26 implants; age: 62.53 ± 7.12 years Control: 10 patients, 38 implants, age: 67.19 ± 9.26 years Diabetic patients were included if HbA1c ≤ 7% Smokers excluded	PD > 5 mm; BOP/suppuration; bone loss	Er, Cr: YSGG laser: 30–80 mJ/pulse, 30–50 Hz	Er, Cr: YSGG laser + OHI (test) MD + OHI (control)	PD (mm): -1.36 ± 1.11 (test)-0.73 ± 0.99 (control) CAL (mm): -1.2 ± 1.43 (test) -0.9 ± 0.91 (control) RBL (mm): 0.05 ± 1.50 (test) 0.02 ± 1.28 (control) BOP (%): -36 ± 47.32 (test) -20 ± 43.14 (control) PI (%): -29 ± 49.27 (test) -30 ± 48.75 (control)	9 M
Roccuzzo [29]	RCT Parallel	25 patients, 25 implants Test: 12 patients, 12 implants; age: 67.3 ± 12.2 years Control: 13 patients, 13 implants; age: 61.0 ± 13.2 years Five patients in the test group and 3 in the control group (*p* = 0.645) were current smokers. (Smoker ≤ 10 cig./day)	PD > 5 mm; BOP/suppuration; RBL ≥ 2 mm	Adjunctive diode laser: 810 nm, 2.5 W, 50 Hz, 10 ms	MD + OHI + adjunctive diode laser (test) MD + OHI (control)	PD (mm): -1.28 ± 0.82 (test) -1.47 ± 0.77 (control) BOP (%): -15.3 ± 31.85 (test) -15.4 ± 25.34 (control) PI: 1.4 ± 15.25 (test) 6.5 ± 24.3 (control) RBL (mm): 0.04 ± 0.98 (test) 0.03 ± 0.54(control)	6 M
Abduljabbar [30]	RCT Parallel	63 patients, 74 implants Test: 31 patients, 35 implants; age: 43.6 ± 6.75 years Control: 32 patients, 39 implants; age: 40.5 ± 7.75 years Smokers excluded	PD ≥ 4 mm and/or bone loss ≥ 3 mm	Nd: YAG laser: 1064 nm 4 W, 80 mJ/pulse, 50 Hz	Nd: YAG laser + MD (test) MD (control)	PD (mm): -2.8 ± 0.35 (test)-1.6 ± 0.47 (control) BOP (%): -39.8 ± 4.97 (test) -39.8 ± 3.68 (control) PI (%): -47.7 ± 3.93 (test) -46 ± 2.49 (control) RBL (mm): 0.1 ± 0.3 (test) -0.1 ± 0.4 (control)	6 M
Arısan [27]	RCT Parallel	10 patients, 48 implants, 55.1 years (range, 43–76; SD, 11.4) Test: 5 patients, 24 implants; Control: 5 patients, 24 implants Smokers excluded	PD: 4–6 mm; BOP/suppuration; MBL < 3 mm	Low-level diode laser (energy density, 3 J/cm^2^; power density, 400 mW/cm^2^; energy, 1.5 J; and spot diameter, 1 mm)	MD + OHI + low-level diode laser (test) MD + OHI (control)	PD (mm): -0.17 ± 0.71 (test) -0.21 ± 0.42 (control) MBL (mm): 0.66 ± 0.48 (test) 0.28 ± 0.55 (control)	6 M
Alqahtani [28]	RCT Parallel	67 patients, 67 implants Test: 34 patients, 34 implants; age: 46.5 ± 3.4 years Control: 33 patients, 33 implants; age: 45.3 ± 1.1 years Smokers excluded	PD ≥ 4 mm; BOP ≥ 30%; CBL ≥ 3 mm	Low-level diode laser: (0.3 W power, 3.41 J/cm^2^, 1.76 cm^2^ spot)	MD + OHI + low-level diode laser (test) MD + OHI (control)	-	6 M
Alpaslan [10]	RCT Parallel	50 patients, 50 implants Er, Cr: YSGG: 17 patients, 17 implants; age: 54.71 ± 7.34 years diode laser: 16 patients, 16 implants age: 46.5 ± 11.34 years control: 17 patients, 17 implants; age: 50.36 ± 6.85 years Smokers excluded	PD: 4–6 mm; BOP /suppuration; bone loss: 2–3 mm	diode laser: (0.8 W power, 3 J/cm^2^, 1 mm spot diameter) Er, Cr: YSGG: (1.5 W power, 140 µs pulse time, 30 Hz frequency, and 1 cm spot size)	MD + diode laser + OHI (diode) MD + OHI + Er, Cr: YSGG laser (Er, Cr: YSGG) MD + OHI (control)	GI: -0.56 ± 0.36 (YSGG) -0.38 ± 0.4 (diode) -0.25 ± 0.28 (control) PI: -0.91 ± 0.33 (YSGG) -0.84 ± 0.63 (diode) -0.64 ± 0.71 (control) PD (mm): -1.16 ± 1.05 (YSGG) -0.86 ± 0.92 (diode) -0.53 ± 0.68 (control) BOP (%): -48.81 ± 19.84 (YSGG) -26.19 ± 25.63 (diode) -11.31 ± 26.91 (control)	6 M
Alpaslan [31]	RCT Parallel	49 patients, 49 implants Test: 23 patients, 23 implants; age: 50 ± 8.81 years control: 26 patients, 26 implants; age: 45.88 ± 9.86 years Smokers excluded	PD: 4–6 mm; MBL: 2–3 mm	Er, Cr: YSGG laser: (1.5 W power, 30 Hz frequency, 140 µs pulse duration, and 1 cm spot size)	MD + OHI + Er, Cr: YSGG laser (tesr) MD + OHI (control)	GI: -0.67 ± 0.47 (test) -0.46 ± 0.48 (control) PI: -0.84 ± 0.45 (test) -0.78 ± 0.64 (control) PD (mm): -1.02 ± 0.82 (test) -0.64 ± 0.75 (control) BOP (%): -39.13 ± 22.16 (test) -27.88 ± 23.71 (control) MBL (mm): -0.026 ± 0.37 (test) 0 ± 0.39 (control)	6 M

Abbreviation: RCT: randomized controlled trial; RBL: radiographic bone loss; PD: probing depth; BOP: bleeding on probing; Er: YAG: erbium-doped: yttrium, aluminum, and garnet; OHI: oral hygiene instructions; MD: mechanical debridement; CHX: Chlorhexidine; CAL: clinical attachment level; GR: gingival recession; PI: plaque index; Nd: YAG: Neodymium-doped yttrium aluminum garnet; Er, Cr: YSGG: erbium, chromium-doped yttrium, scandium, gallium and garnet; MBL: marginal bone loss; CBL: crestal bone loss; GI: gingival index

### Risk of bias

**Fig. 2:**
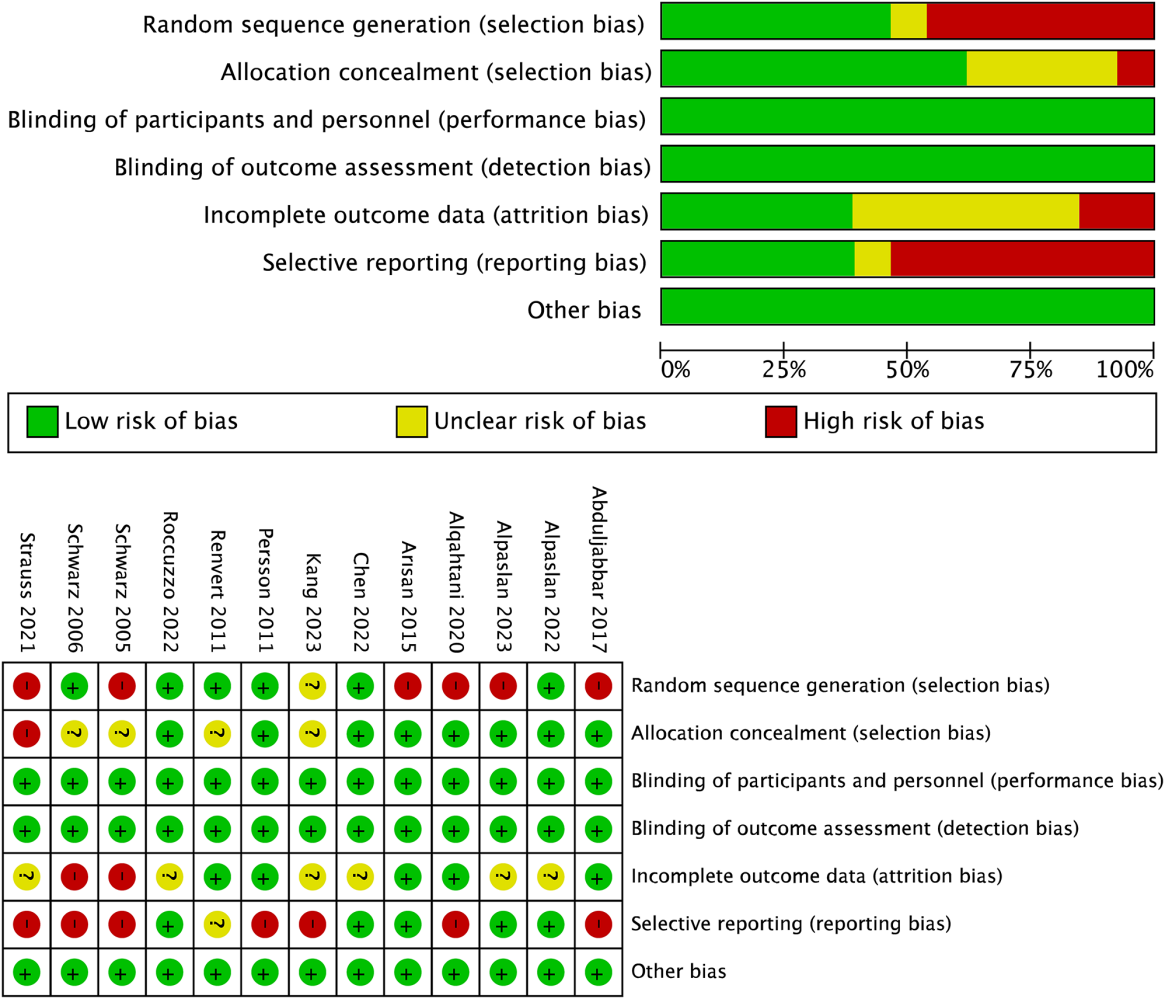
Quality assessment of the selected studies (the Revised Cochrane risk of bias tool for randomized trials (rob2)). Green represents low risk of bias, yellow represents unclear risk of bias and red represents a high risk of bias

Because of the implementation of a coin toss for participant grouping, five studies have been identified with a high risk of bias [24, 27, 28, 30, 31]. Furthermore, the practice of alternating enrollment assignments based on the timing of registration had similarly been flagged for high risk of bias [22]. Withdrawals occurred in eight trials [10, 11, 21–24, 29, 31], two of these trials were judged at high risk of bias [23, 24]. In the selective reporting (reporting bias), six trials were judged to be at low risk of bias, one at unclear risk of bias whereas all the remaining trials were judged to be at high risk of bias because standard deviations were not reported (Fig. 2) [11, 22–24, 26, 28, 30].

### Grade assessment

The GRADE assessment (Tab. S1-5) evaluated the efficacy of laser treatments for peri-implantitis in comparison to control treatments. The clinical outcomes assessed include PD, bone loss, BOP, CAL, and PI across various laser types (diode, Er: YAG, Er, Cr: YSGG and Nd: YAG). Except for bone loss, the other outcomes demonstrated evidence with moderate certainty. Subsequently, subgroup analysis revealed several issues affecting the certainty of evidence. High reporting bias and attrition bias contributed to a serious or very serious risk of bias. Additionally, the small sample size and wide confidence intervals for the outcomes further complicated the assessment. As a result, the certainty of evidence for the following outcomes was rated as very low: PD (Er: YAG), bone loss (Nd: YAG), BOP (Er: YAG), CAL (Er: YAG), and PI (Er: YAG). For the majority of the remaining subgroup outcomes, the certainty of evidence was rated as moderate.

### Study outcomes

#### Primary outcomes

With regard to PD, four studies reported a significant difference (*P* < 0.5) between laser group and control group [10, 11, 23, 31]. In contrast, remaining studies did not show any difference [21, 22, 24–27, 29, 30].

#### Secondary outcomes

With regard to bone loss, seven studies were evaluated the changes between the experimental group and control group. Only two studies demonstrated a significant difference, indicating that bone loss increased after laser treatment [27, 31]. Three studies reported a significant difference in BOP between the experimental and control group [23, 24, 31]. For PI, the study from schwarz et al. showed that there was a significant difference in the experimental group compared with the control group at 12 months [23]. Regarding CAL, only one study reported a significant difference between the experimental group and control group at 6 months [23].

### Meta-analysis

**Fig. 3:**
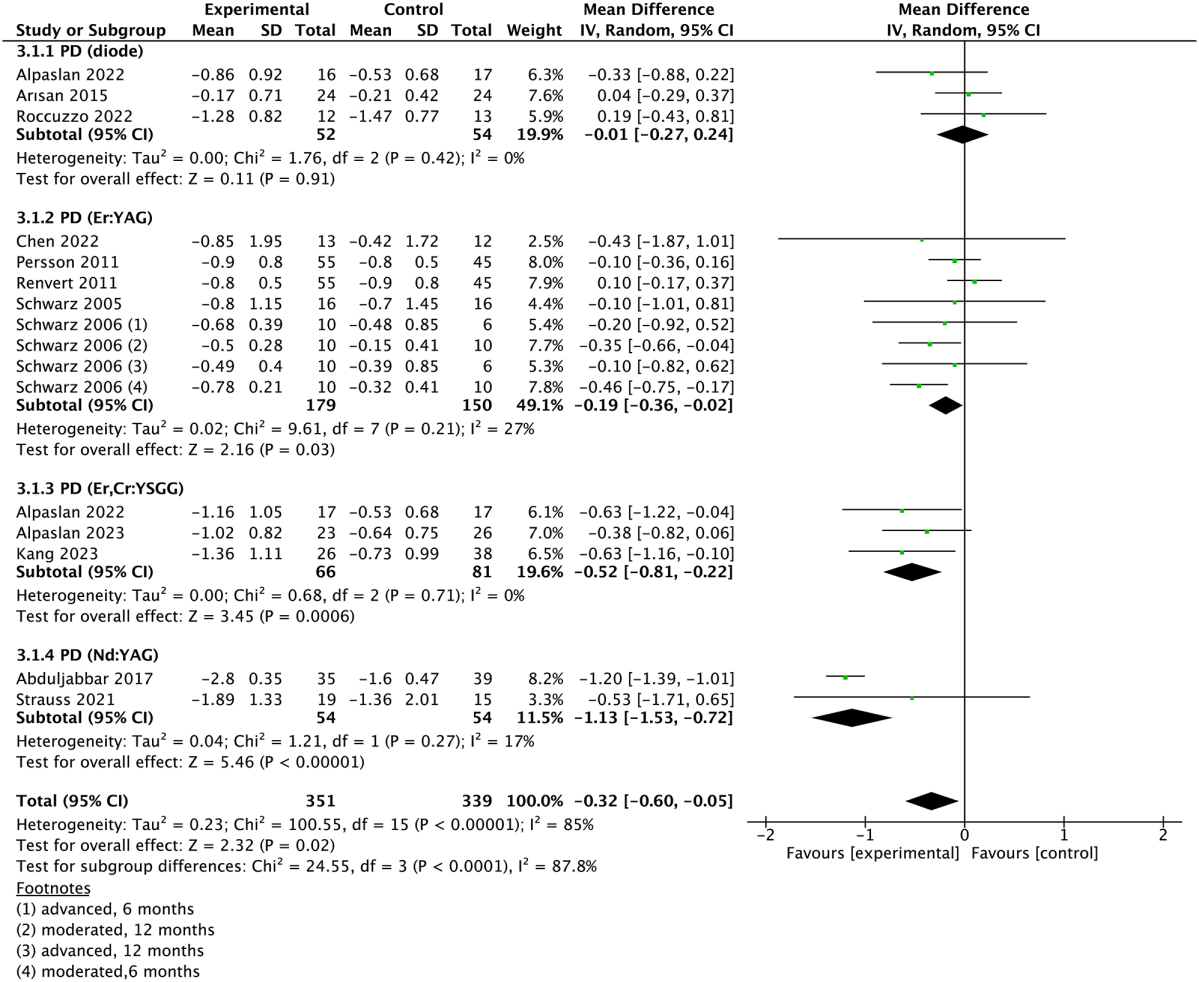
Forest plot of PD changes at implant level

**Fig. 4:**
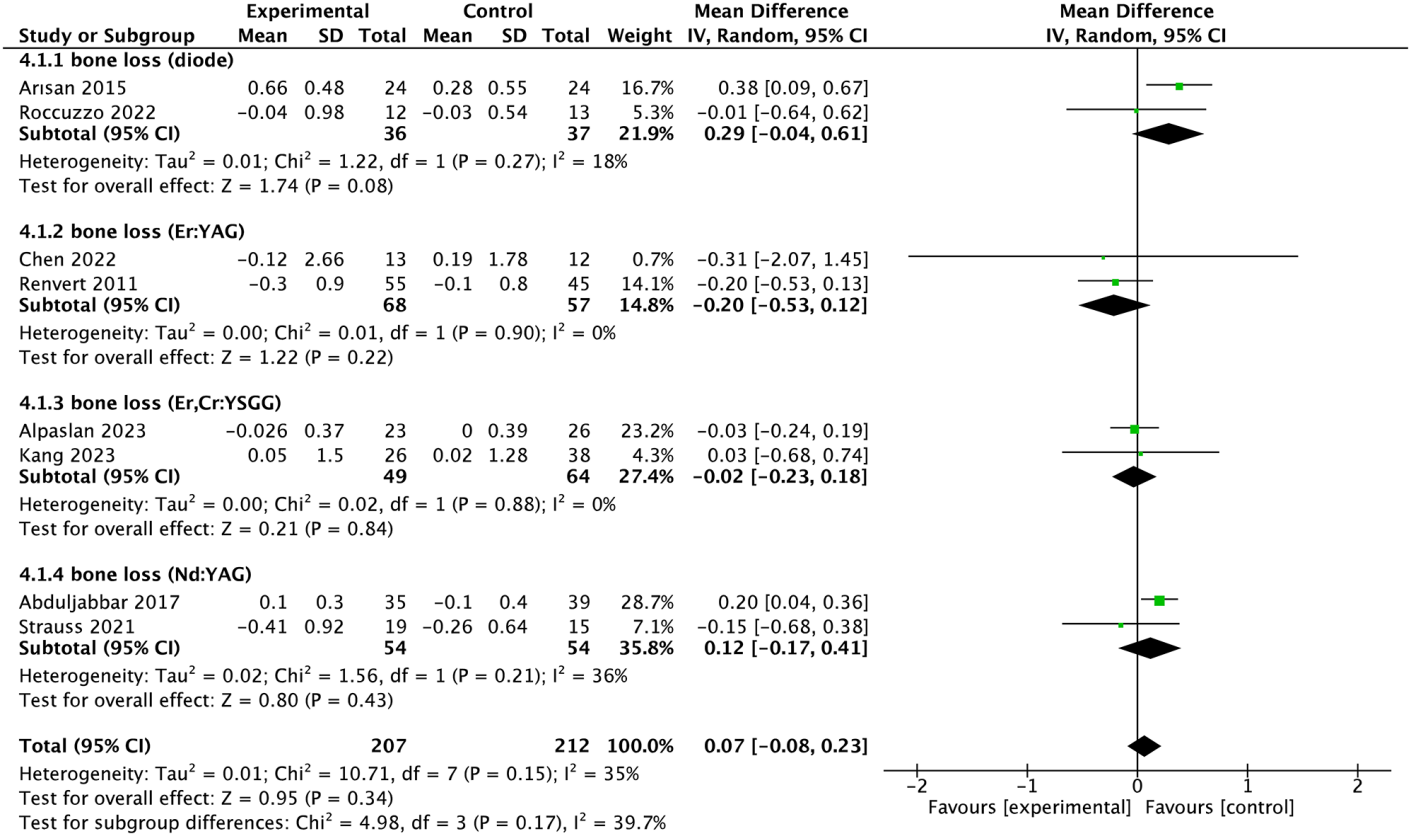
Forest plot of bone loss changes at implant level

**Fig. 5:**
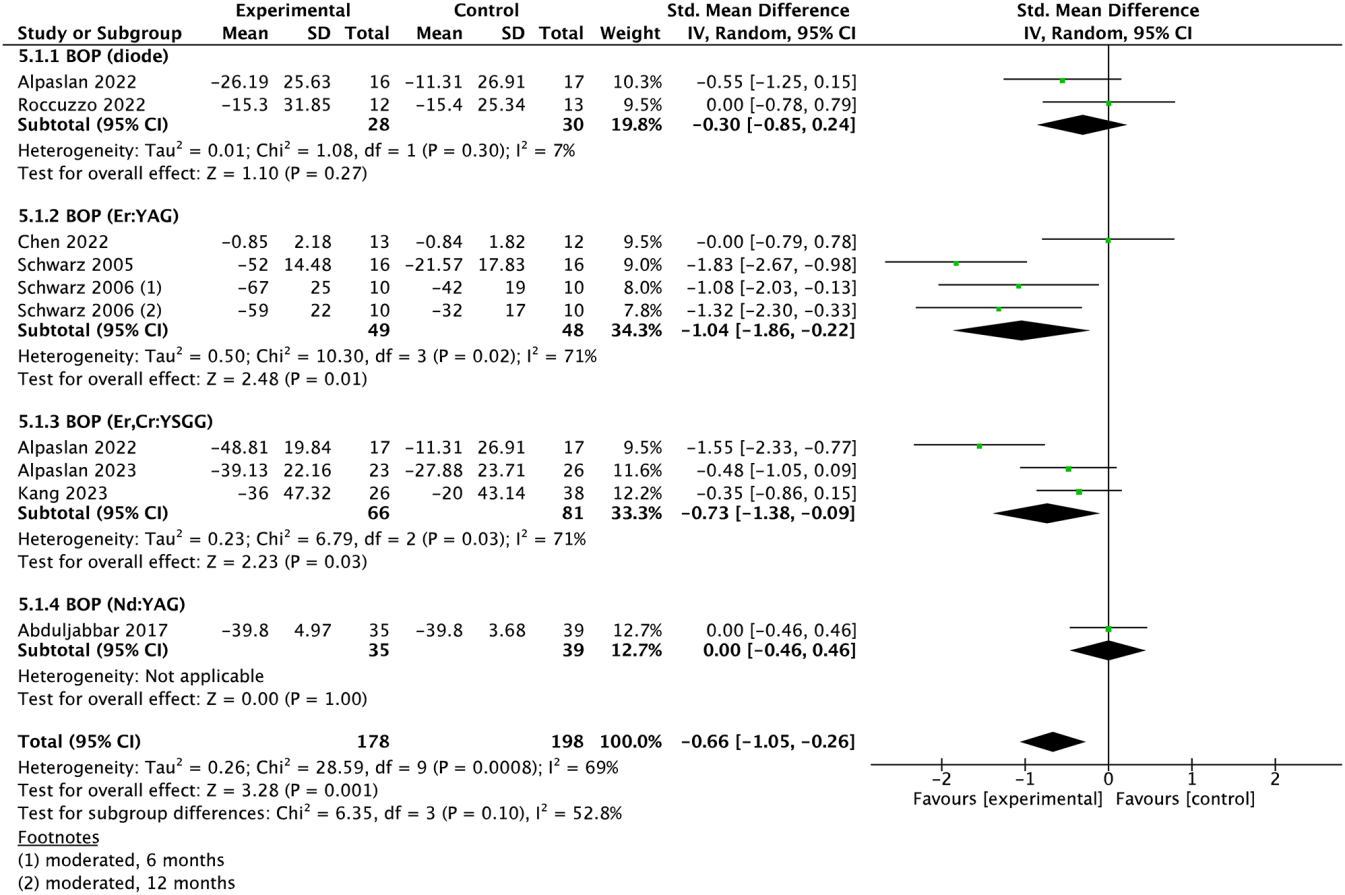
Forest plot of BOP changes at implant level

**Fig. 6:**
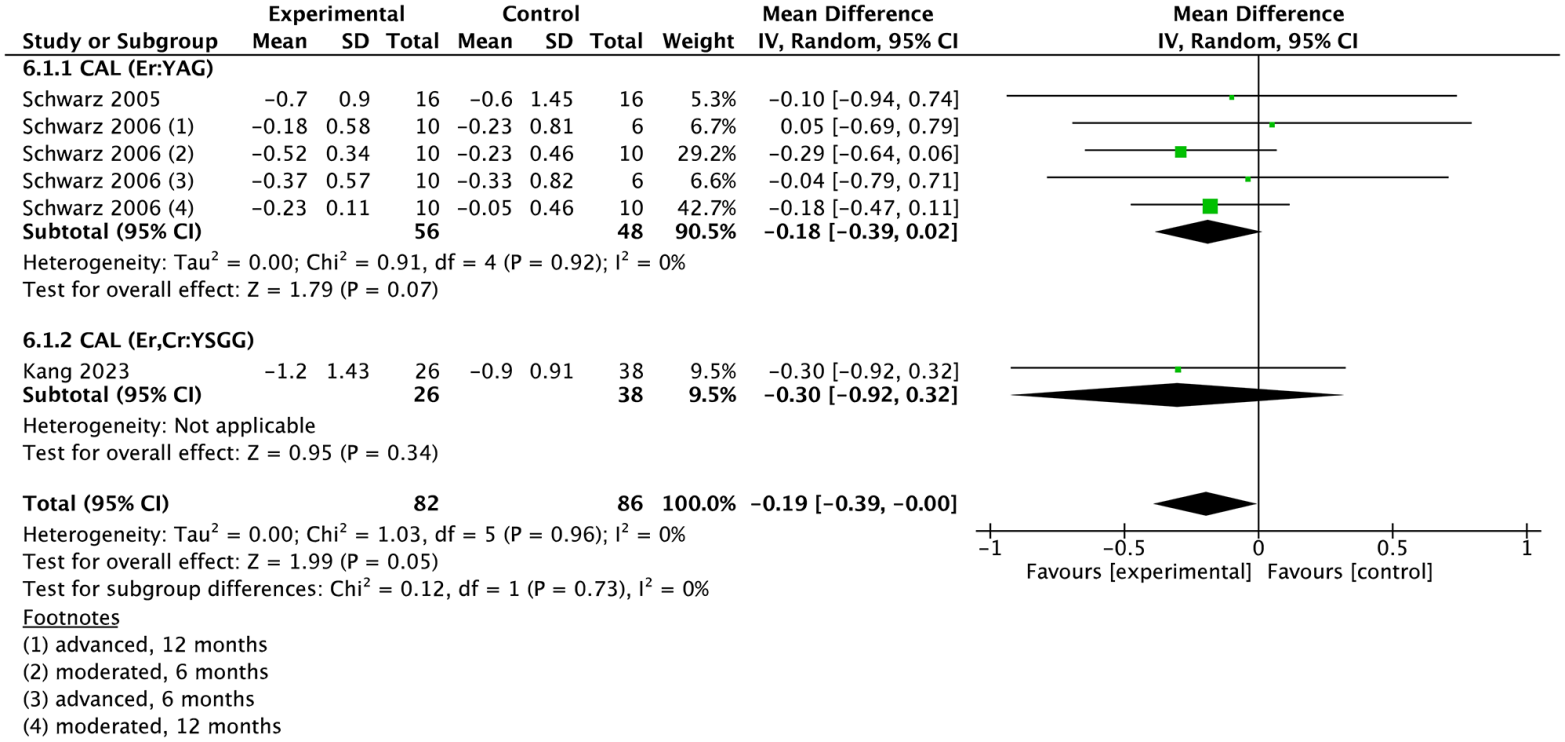
Forest plot of CAL changes at implant level

**Fig. 7:**
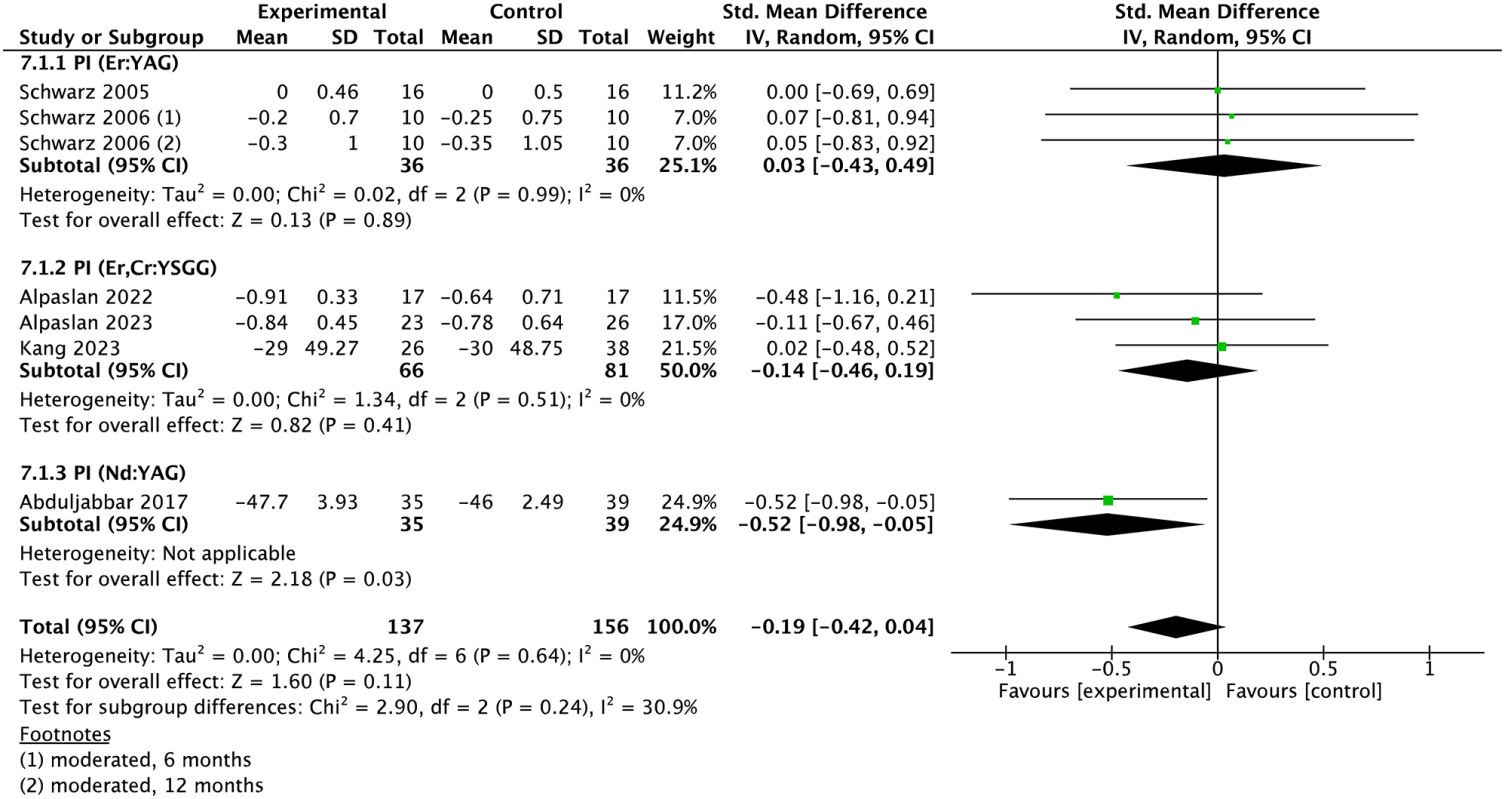
Forest plot of PI changes at implant level

A meta-analysis was conducted on studies with similar clinical outcomes of PD, bone loss, BOP, PI and CAL. Based on included studies, the overall WMD in PD between the experimental and control group was − 0.32 [95% CI (-0.60, -0.05), p = 0.02]. P value (< 0.1) and I^2^ value (85%) for heterogeneity of PD changes indicated substantial heterogeneity (Fig. 3). Therefore, the subgroup analysis was performed according to the types of lasers used, and the result demonstrated that the heterogeneity of each type laser was low (Fig. 3). What’ more, the WMD in PD changes favored the solid-state lasers instead of diode laser in the experimental group compared with control group (Fig. S1). The meta-analysis failed to show a significant bone loss changes (WMD = 0.07, 95% CI -0.08 to 0.23, *p* = 0.34) (Fig. 4). The overall SMD in BOP between the two groups was − 0.66 [95% CI (-1.05, -0.26), *p* = 0.001], which suggests that BOP significantly decreased in patients with peri-implantitis using laser therapy. There was a substantial heterogeneity (*p* = 0.0008, I^2^ = 69%). Further subgroup analysis indicated that Er: YAG and Er, Cr: YSGG lasers were particularly effective in reducing BOP compared to other laser types(Fig. 5). The changes in CAL were shown in Fig. 6. The WMD of CAL between the two groups was − 0.19 (95% CI (− 0.39, − 0.00), *p* = 0.05), indicating a significant decrease in CAL for patients with peri-implantitis treated with laser therapy, with low heterogeneity(*p* = 0.96; I^2^ = 0%). Lastly, the SMD in PI between the two groups was − 0.19 [95% CI (-0.42, 0.04), *p* = 0.11], suggesting that PI didn’t significantly decrease in patients with peri-implantitis using laser therapy (*p* = 0.64, I^2^ = 0%, low heterogeneity) (Fig. 7).

### Publication bias

In this systematic review and meta-analysis, we found no evidence of publication bias by the result of the Egger’s tests (*p* > 0.05) (Fig. S2).

### Sensitivity analysis

Sensitivity analysis (leave-one-out method) revealed no significant change in the pooled estimation when excluding any individual study (Fig. S3).

## Discussion

### Summary of findings

The findings of this system review and meta-analysis showed that the solid-state lasers (Er: YAG, Er, Cr: YSGG and Nd: YAG laser) markedly improved PD, BOP and CAL in comparison to the control group. Regardless of the type of laser used between the experimental and control group, no noticeable differences were observed in laser therapy for bone loss and PI. Moreover, the available evidence at present didn’t support any significant differences about the clinical efficacy of diode laser. In our study, one article was excluded from the meta-analysis as the clinical data could not be obtained, even after reaching out to the authors [28]. The heterogeneity of the outcomes may be explained by the variations in the types of lasers used, as revealed through subgroup analysis. No evidence indicated the existence of publication bias. The sensitivity analysis (leave-one-out) confirmed that the pooled estimate was stable. Through the GRADE assessment, all outcomes except bone loss demonstrated evidence with moderate certainty.

### Agreements and disagreements with other studies

Recent studies have shed light on the limitations of CO_2_ and diode lasers in effectively removing plaque from titanium implants, aligning with the results of our own meta-analysis [13, 14]. Consequently, these lasers were primarily employed as supplementary procedures. Nd: YAG lasers, possessing high peak power and a moderate reflection rate from titanium, have the potential to ablate titanium but may also pose a risk to implant surfaces [32, 33]. Abduljabbar et al. reported a 0.1 mm decrease in bone level following Nd: YAG laser treatment, potentially attributed to implant surface damage. By contrast, in vitro studies utilizing Nd: YAG lasers with longer pulse durations and non-contact modes showed no damage to titanium surfaces [34]. Given the limited current research on this laser, it is imperative to exercise caution when considering the use of Nd: YAG lasers, paying close attention to irradiation parameters and the laser beam’s location [35].

With regard to BOP, there were conflicting findings in the studies included in our review [10, 11, 21, 23, 24, 29–31]. Schwarz et al. reported a statistically significant reduction in BOP after Er: YAG laser irradiation [23, 24]. This effect could be attributed to tissue coagulation or vaporization following laser treatment. In contrast, Chen et al. found that the control group exhibited a significant reduction in anaerobic bacterial counts at the 3-month and 6-month follow-up periods. However, this reduction did not translate into significant differences in the improvement of BOP reduction [21]. Badersten et al. pointed out that measurements around implants can potentially traumatize tissues, leading to significant bleeding [36]. The healing of periodontal tissues may take place during the 9–12 months after treatment. These findings emphasized that long-term control of tissue inflammation may be more closely associated with a maintenance protocol rather than the active treatment itself. Therefore, clinicians should exercise caution when interpreting the presence of BOP, as it may not accurately reflect the inflammatory status of peri-implant tissues.

Interestingly, our meta-analysis results suggested that patients with deeper pockets were likely to experience more significant changes in PD and CAL, in alignment with previous studies [37]. This connection between PD changes following peri-implantitis treatment and baseline PD is noteworthy.

In the context of bone loss, this meta-analysis did not find significant evidence of a change within the laser treatment group when compared to the control group. Some studies typically assessed interventions at follow-up periods of 3, 6, 9, or twelve months [38, 39]. A 6-month period for assessing bone changes through intraoral radiographs is relatively short, which may pose challenges in detecting differences between the baseline and the 6-month follow-up, consistent with our findings [25]. Additionally, Mombelli et al. suggested that surgical intervention might be necessary for considerable bone destruction (bone loss > 3 mm) to correct tissue morphology or employ guided bone regeneration techniques [40]. However, the grading of bone loss wasn’t uniform in some studies, preventing confirmation of this statement. Therefore, further research is essential to ascertain the necessity of surgical intervention based on the extent of bone loss and the severity of peri-implantitis. Adopting such a tailored approach could yield more scientifically validated and efficacious treatments, potentially shortening treatment duration and enhancing patient outcomes.

It is noteworthy that in the currently published literature within this field, almost none of the articles mention the period between experimental/control treatment to the assessment of treatment outcomes. Most of the included studies provided information on OHI, with only two studies not addressing this aspect [21, 30]. During the follow-up period, several studies reported different degrees of retreatment or multiple interventions [11, 21–24, 29]. Additionally, some studies provided patients with toothbrushes or interdental brushes [25, 26, 29]. Consequently, differences in treatment modalities and follow-up frequencies among studies could significantly impact the prognosis and analysis outcomes, thereby necessitating additional research for validation.

Finally, we compared the relevant systematic reviews and meta-analysis of this aspect in recent years. In 2018, Lin et al. conducted a meta-analysis assessing the efficacy of laser therapy as an adjunctive treatment modality, in conjunction with surgical and non-surgical interventions, for the treatment of peri-implantitis and peri-implant mucositis [41]. Since then, several high-quality RCTs had further explored the potential of laser therapy in treating peri-implantitis, both as an adjunctive and standalone treatment option. Although recent systematic reviews have incorporated some of these new RCTs, no meta-analyses have yet been conducted in these reviews [42, 43]. And the latest review included the articles published before July 2021, in which the study objects have laser or photodynamic therapy [43]. In 2023, Barbato et al. performed a meta-analysis to evaluate a range of non-surgical interventions for peri-implantitis, encompassing PDT, laser therapy, and systemic antibiotics [44]. Notably, their results did not conclusively endorse any specific treatment modality, likely due to the diversity of interventions studied. Therefore, a meta-analysis on the efficacy of laser in the nonsurgical treatment of peri-implantitis is needed.

### Limitation

In this systematic review and meta-analysis, some limitations arise from the inevitable differences among included studies. Firstly, as smoking has been proven to be a risk factor of peri-implantitis, only nine studies excluded smoker patients, while the other three studies included both smokers and nonsmokers. It may potentially jeopardize the results of pooled estimates. Secondly, the inclusion of different stages of peri-implantitis (moderated and advanced stages) in some studies complicated assessing laser effects at specific stages. Unfortunately, due to limited data and clinical mixing, subgroup analysis was not feasible. Thirdly, variations in diagnostic criteria, patient adherence to post-treatment care, laser parameters, implant surfaces, and implant superstructures could significantly influence outcomes. The design and characteristics of implant superstructures may particularly affect cleanability and the risk of peri-implantitis. Finally, limited data, high heterogeneity, and small-scale studies may slightly diminish the quality and impact of the conclusions in this systematic review.

## Conclusion

In the non-surgical treatment of peri-implantitis, solid-state lasers yielded positive influence on the peri-implantitis healing in term of improving PD, BOP and CAL, while diode laser provided no beneficial effect. Considering this conclusion and the limitations inherent in the included studies, it is imperative for future researches to encompass extended patient follow-up periods and to undertake rigorous, multi-center, large-sample randomized controlled trials to ensure high-quality outcomes.

## Electronic supplementary material

Below is the link to the electronic supplementary material.

Supplementary Material 1

Supplementary Material 2: Tab. S1-5: GRADE assessment

Supplementary Material 3: Fig. S1: Forest plot of changes in PD, bone loss, and BOP at implant level for the treatment of solid-state laser.

Supplementary Material 4: Fig. S2: The result of Egger’s test. (A) PD; (B) bone loss; (C) BOP; (D) CAL; (E) PI.

Supplementary Material 5: Fig. S3: Sensitivity analysis of primary and secondary outcomes. (A) PD; (B) bone loss; (C) BOP; (D) CAL; (E) PI.

## Data Availability

No datasets were generated or analysed during the current study.
