# Supplementary material for: The clinical efficacy of laser in the nonsurgical treatment of peri-implantitis: a systematic review and meta-analysis
Source: Int J Implant Dent. 2024 Nov 14;10:54. doi: 10.1186/s40729-024-00570-x (PMC11564455; doi:10.1186/s40729-024-00570-x)
Supplement: Supplementary file 1 — Supplementary Material 1 [file 40729_2024_570_MOESM1_ESM.docx]

**Supplementary file**

1. PubMed

| Search | Query | Items found |
| --- | --- | --- |
| #1 | ((((laser therapy[MeSH Terms])) OR (laser[MeSH Terms])) OR (laser therapy[Text Word])) OR (laser[Text Word]) | 370684 |
| #2 | ((((peri-implantitis[MeSH Terms]) OR (peri-implant infections[Text Word])) OR (peri-implantitis[Text Word])) OR (peri-implant bone loss[Text Word])) OR (peri-implant defect[Text Word]) OR (peri-implant bone loss[Text Word]) | 4974 |
| #3 | #1 and #2 | 428 |

1. Web of science

| Search | Query | Items found |
| --- | --- | --- |
| #1 | (TS=(laser)) OR TS=(laser therapy) | 547471 |
| #2 | ((((TS=(peri-implantitis )) OR TS=(peri-implant bone loss)) OR TS=(peri-implant tissue loss)) OR TS=(peri-implant defect)) OR TS=(peri-implant infections) | 6158 |
| #3 | #1 and #2 | 588 |

1. Cochrane Central Register of Controlled Trials (CENTRAL) search strategy

| Search | Query | Items found |
| --- | --- | --- |
| #1 | MeSH descriptor: [Peri-Implantitis] explode all trees | 363 |
| #2 | peri-implant infections | 45 |
| #3 | peri-implant bone loss | 921 |
| #4 | peri-implant tissue loss | 418 |
| #5 | peri-implant defect | 148 |
| #6 | #1 or #2 or #3 or #4 or #5 | 1285 |
| #7 | MeSH descriptor: [Lasers] explode all trees | 3807 |
| #8 | laser therapy | 14864 |
| #9 | #7 or #8 | 16241 |
| #10 | #6 and #9 | 80 |

1. Scopus

| Search | Query | Items found |
| --- | --- | --- |
| #1 | ( ALL ( laser AND therapy OR laser ) AND ALL ( peri-implantitis OR peri-implant AND infections OR peri-implantitis OR peri-implant AND bone AND loss OR peri-implant AND defect )) AND (LIMIT-TO ( LANGUAGE , "English" ) ) | 2699 |
