# Supplementary material for: The clinical efficacy of laser in the nonsurgical treatment of peri-implantitis: a systematic review and meta-analysis
Source: Int J Implant Dent. 2024 Nov 14;10:54. doi: 10.1186/s40729-024-00570-x (PMC11564455; doi:10.1186/s40729-024-00570-x)
Supplement: Supplementary file 2 — Supplementary Material 2: Tab. S1-5: GRADE assessment [file 40729_2024_570_MOESM2_ESM.docx]

Table S1. GRADE assessment. Laser treatment compared to control treatment for PD of peri-implantitis

| **Certainty assessment** | | | | | | | **№ of patients** | | **Effect** | | **Certainty** | **Importance** |
| --- | --- | --- | --- | --- | --- | --- | --- | --- | --- | --- | --- | --- |
| **№ of studies** | **Study design** | **Risk of bias** | **Inconsistency** | **Indirectness** | **Imprecision** | **Other considerations** | **laser treatment** | **control treatment** | **Relative (95% CI)** | **Absolute (95% CI)** |  |  |
| **PD (all)** | | | | | | | | | | | | |
| 12 | randomised trials | serious^a^ | not serious | not serious | not serious | none | 351 | 339 | - | MD **0.32 lower** (0.6 lower to 0.05 lower) | ⨁⨁⨁◯ Moderate^a^ | CRITICAL |
| **PD (diode)** | | | | | | | | | | | | |
| 3 | randomised trials | not serious | not serious | not serious | serious^b^ | none | 52 | 54 | - | MD **0.01 lower** (0.27 lower to 0.24 higher) | ⨁⨁⨁◯ Moderate^b^ | CRITICAL |
| **PD (Er:YAG)** | | | | | | | | | | | | |
| 5 | randomised trials | very serious^c,d^ | not serious | not serious | serious^b^ | none | 179 | 150 | - | MD **0.19 lower** (0.36 lower to 0.02 lower) | ⨁◯◯◯ Very low^b,c,d^ | CRITICAL |
| **PD (Er,Cr:YSGG)** | | | | | | | | | | | | |
| 3 | randomised trials | serious^e,f^ | not serious | not serious | not serious | none | 66 | 81 | - | MD **0.52 lower** (0.81 lower to 0.22 lower) | ⨁⨁⨁◯ Moderate^e,f^ | CRITICAL |
| **PD (Nd:YAG)** | | | | | | | | | | | | |
| 2 | randomised trials | serious^g^ | not serious | not serious | not serious | none | 54 | 54 | - | MD **1.13 lower** (1.53 lower to 0.72 lower) | ⨁⨁⨁◯ Moderate^g^ | CRITICAL |

**CI:** confidence interval; **MD:** mean difference

#### Explanations

a. Among the 12 articles included, 5 had high selection bias and 6 had high reporting bias. b. The sample size was small and the confidence interval of the outcome was wide. c. Three of the studies had high reporting bias. d. Two of the studies had high attrition bias. e. One of the studies had high reporting bias. f. One of studies had high selection bias. g. Two of studies had high selection bias and reporting bias.

Table S2. GRADE assessment. Laser treatment compared to control treatment for bone loss of peri-implantitis

| **Certainty assessment** | | | | | | | **№ of patients** | | **Effect** | | **Certainty** | **Importance** |
| --- | --- | --- | --- | --- | --- | --- | --- | --- | --- | --- | --- | --- |
| **№ of studies** | **Study design** | **Risk of bias** | **Inconsistency** | **Indirectness** | **Imprecision** | **Other considerations** | **laser treatment** | **control treatment** | **Relative (95% CI)** | **Absolute (95% CI)** |  |  |
| **bone loss (all)** | | | | | | | | | | | | |
| 8 | randomised trials | serious^a^ | not serious | not serious | serious^b^ | none | 207 | 212 | - | MD **0.07 higher** (0.08 lower to 0.23 higher) | ⨁⨁◯◯ Low^a,b^ | IMPORTANT |
| **bone loss(diode)** | | | | | | | | | | | | |
| 2 | randomised trials | not serious | not serious | not serious | serious^c^ | none | 36 | 37 | - | MD **0.29 higher** (0.04 lower to 0.61 higher) | ⨁⨁⨁◯ Moderate^c^ | IMPORTANT |
| **bone loss (Er:YAG)** | | | | | | | | | | | | |
| 2 | randomised trials | not serious | not serious | not serious | serious^c^ | none | 68 | 57 | - | MD **0.2 lower** (0.53 lower to 0.12 higher) | ⨁⨁⨁◯ Moderate^c^ | IMPORTANT |
| **bone loss (Er,Cr:YSGG)** | | | | | | | | | | | | |
| 2 | randomised trials | serious^d^ | not serious | not serious | serious^c^ | none | 49 | 64 | - | MD **0.02 lower** (0.23 lower to 0.18 higher) | ⨁⨁◯◯ Low^c,d^ | IMPORTANT |
| **bone loss (Nd:YAG)** | | | | | | | | | | | | |
| 2 | randomised trials | very serious^e^ | not serious | not serious | serious^c^ | none | 54 | 54 | - | MD **0.12 higher** (0.17 lower to 0.41 higher) | ⨁◯◯◯ Very low^c,e^ | IMPORTANT |

**CI:** confidence interval; **MD:** mean difference

#### Explanations

a. Among the 8 articles included, 3 had high selection bias and 2 had high reporting bias. b. 95% CI was consistent with the possibility for important benefit and large harm exceeding a minimal important difference c. The sample size was small and the confidence interval of the outcome was wide. d. One study had high reporting bias, and the other study had high selection bias. e. Both studies had high reporting bias and selection bias.

Table S3. GRADE assessment. Laser treatment compared to control treatment for BOP of peri-implantitis

| **Certainty assessment** | | | | | | | **№ of patients** | | **Effect** | | **Certainty** | **Importance** |
| --- | --- | --- | --- | --- | --- | --- | --- | --- | --- | --- | --- | --- |
| **№ of studies** | **Study design** | **Risk of bias** | **Inconsistency** | **Indirectness** | **Imprecision** | **Other considerations** | **laser treatment** | **control treatment** | **Relative (95% CI)** | **Absolute (95% CI)** |  |  |
| **BOP (all)** | | | | | | | | | | | | |
| 8 | randomised trials | serious^a^ | not serious | not serious | not serious | none | 178 | 198 | - | SMD **0.66 lower** (1.05 lower to 0.26 lower) | ⨁⨁⨁◯ Moderate^a^ | CRITICAL |
| **BOP (diode)** | | | | | | | | | | | | |
| 2 | randomised trials | not serious | not serious | not serious | not serious | none | 28 | 30 | - | SMD **0.3 lower** (0.85 lower to 0.24 higher) | ⨁⨁⨁⨁ High | CRITICAL |
| **BOP (Er:YAG)** | | | | | | | | | | | | |
| 3 | randomised trials | very serious^b^ | serious^c^ | not serious | not serious | none | 49 | 48 | - | SMD **1.04 lower** (1.86 lower to 0.22 lower) | ⨁◯◯◯ Very low^b,c^ | CRITICAL |
| **BOP (Er,Cr:YSGG)** | | | | | | | | | | | | |
| 3 | randomised trials | serious^d^ | serious^c^ | not serious | not serious | none | 66 | 81 | - | SMD **0.73 lower** (1.38 lower to 0.09 lower) | ⨁⨁◯◯ Low^c,d^ | CRITICAL |
| **BOP (Nd:YAG)** | | | | | | | | | | | | |
| 1 | randomised trials | serious^e^ | not serious | not serious | not serious | none | 35 | 39 | - | SMD **0**  (0.46 lower to 0.46 higher) | ⨁⨁⨁◯ Moderate^e^ | CRITICAL |

**CI:** confidence interval; **SMD:** standardised mean difference

#### Explanations

a. Among the 8 articles included, 3 had high selection bias, 2 had attrition bias and 4 had high reporting bias. b. Two of studies had high reporting bias and attrition bias, and one of studies had high selection bias. c. There was serious heterogeneity among the studies, and no reasonable explanation was found. d. One of studies had high selection bias. e. The study had high selection bias and reporting bias.

Table S4. GRADE assessment. Laser treatment compared to control treatment for CAL of peri-implantitis

| **Certainty assessment** | | | | | | | **№ of patients** | | **Effect** | | **Certainty** | **Importance** |
| --- | --- | --- | --- | --- | --- | --- | --- | --- | --- | --- | --- | --- |
| **№ of studies** | **Study design** | **Risk of bias** | **Inconsistency** | **Indirectness** | **Imprecision** | **Other considerations** | **laser treatment** | **control treatment** | **Relative (95% CI)** | **Absolute (95% CI)** |  |  |
| **CAL (all)** | | | | | | | | | | | | |
| 3 | randomised trials | serious^a^ | not serious | not serious | not serious | none | 82 | 86 | - | MD **0.19 lower** (0.39 lower to 0 ) | ⨁⨁⨁◯ Moderate^a^ | IMPORTANT |
| **CAL (Er:YAG)** | | | | | | | | | | | | |
| 2 | randomised trials | very serious^b^ | not serious | not serious | serious^c^ | none | 56 | 48 | - | MD **0.18 lower** (0.39 lower to 0.02 higher) | ⨁◯◯◯ Very low^b,c^ | IMPORTANT |
| **CAL (Er,Cr:YSGG)** | | | | | | | | | | | | |
| 1 | randomised trials | not serious | not serious | not serious | not serious | none | 26 | 38 | - | MD **0.3 lower** (0.92 lower to 0.32 higher) | ⨁⨁⨁⨁ High | IMPORTANT |

**CI:** confidence interval; **MD:** mean difference

#### Explanations

a. Among the 3 articles included, 3 had high selection bias, 2 had attrition bias and 1 had high reporting bias. b. Two of the studies had high reporting bias and attrition bias, and one of the studies had selection bias. c. The sample size was small and the confidence interval of the outcome was wide.

Table S5. GRADE assessment. Laser treatment compared to control treatment for PI of peri-implantitis

| **Certainty assessment** | | | | | | | **№ of patients** | | **Effect** | | **Certainty** | **Importance** |
| --- | --- | --- | --- | --- | --- | --- | --- | --- | --- | --- | --- | --- |
| **№ of studies** | **Study design** | **Risk of bias** | **Inconsistency** | **Indirectness** | **Imprecision** | **Other considerations** | **laser treatment** | **control treatment** | **Relative (95% CI)** | **Absolute (95% CI)** |  |  |
| **PI (all)** | | | | | | | | | | | | |
| 6 | randomised trials | serious^a^ | not serious | not serious | not serious | none | 137 | 156 | - | SMD **0.19 lower** (0.42 lower to 0.04 higher) | ⨁⨁⨁◯ Moderate^a^ | IMPORTANT |
| **PI (Er:YAG)** | | | | | | | | | | | | |
| 2 | randomised trials | very serious^b^ | not serious | not serious | serious^c^ | none | 36 | 36 | - | SMD **0.03 higher** (0.43 lower to 0.49 higher) | ⨁◯◯◯ Very low^b,c^ | IMPORTANT |
| **PI (Er,Cr:YSGG)** | | | | | | | | | | | | |
| 3 | randomised trials | serious^d^ | not serious | not serious | not serious | none | 66 | 81 | - | SMD **0.14 lower** (0.46 lower to 0.19 higher) | ⨁⨁⨁◯ Moderate^d^ | IMPORTANT |
| **PI (Nd:YAG)** | | | | | | | | | | | | |
| 1 | randomised trials | serious^e^ | not serious | not serious | not serious | none | 35 | 39 | - | SMD **0.52 lower** (0.98 lower to 0.05 lower) | ⨁⨁⨁◯ Moderate^e^ | IMPORTANT |

**CI:** confidence interval; **SMD:** standardised mean difference

#### Explanations

a. Among the 6 articles included, 4 had high selection bias, 2 had attrition bias and 3 had high reporting bias. b. Two of the studies had high reporting bias and attrition bias, and one of the studies had selection bias. c. The sample size was small and the confidence interval of the outcome was wide. d. One of studies had high selection bias. e. The study had high selection bias and reporting bias.
