# Supplementary figures and images for: The clinical efficacy of laser in the nonsurgical treatment of peri-implantitis: a systematic review and meta-analysis
Source: Int J Implant Dent. 2024 Nov 14;10:54. doi: 10.1186/s40729-024-00570-x (PMC11564455; doi:10.1186/s40729-024-00570-x)

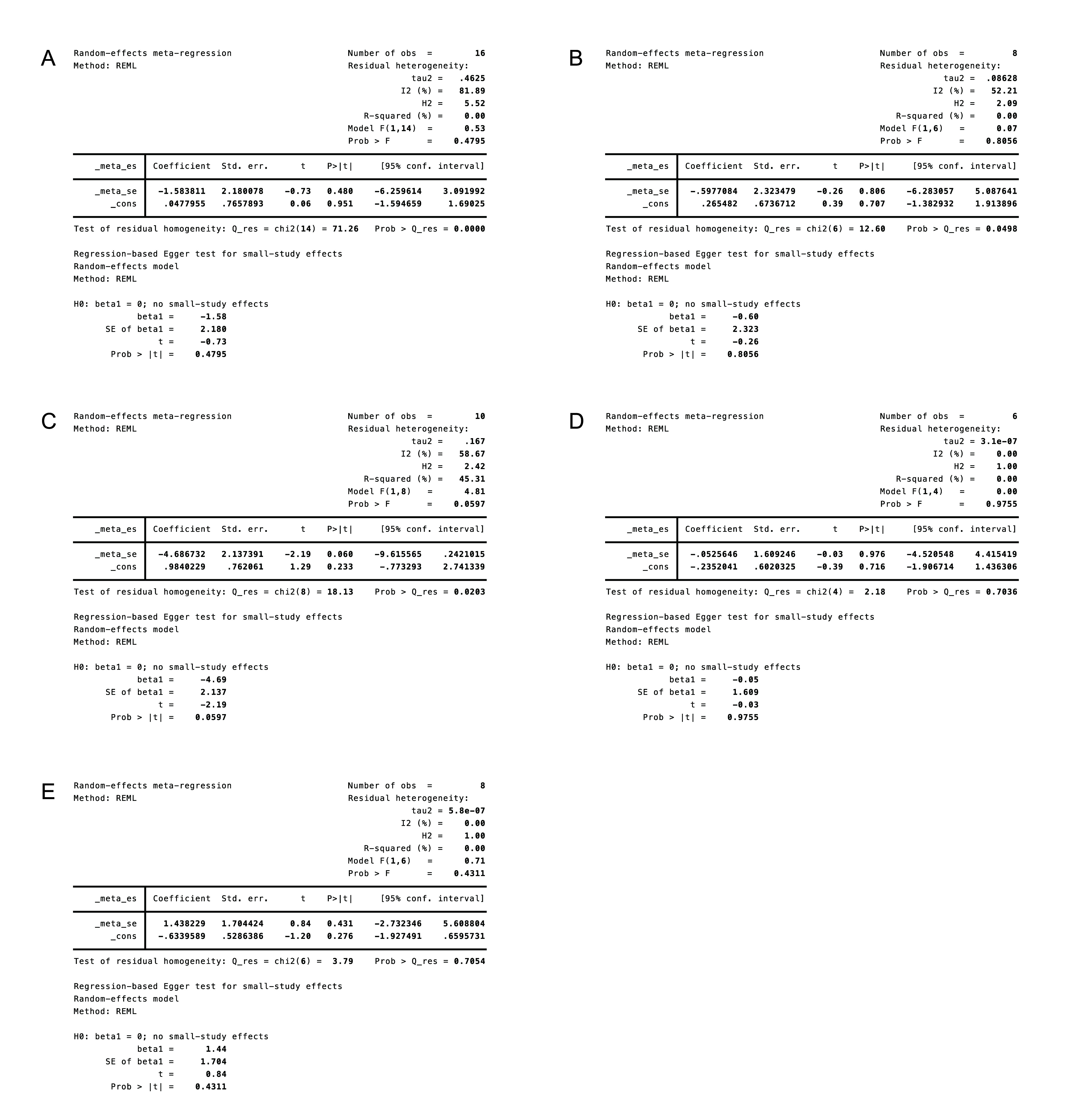

Supplement: Supplementary file 4 — Supplementary Material 4: Fig. S2: The result of Egger’s test. (A) PD; (B) bone loss; (C) BOP; (D) CAL; (E) PI. [file 40729_2024_570_MOESM4_ESM.tif]

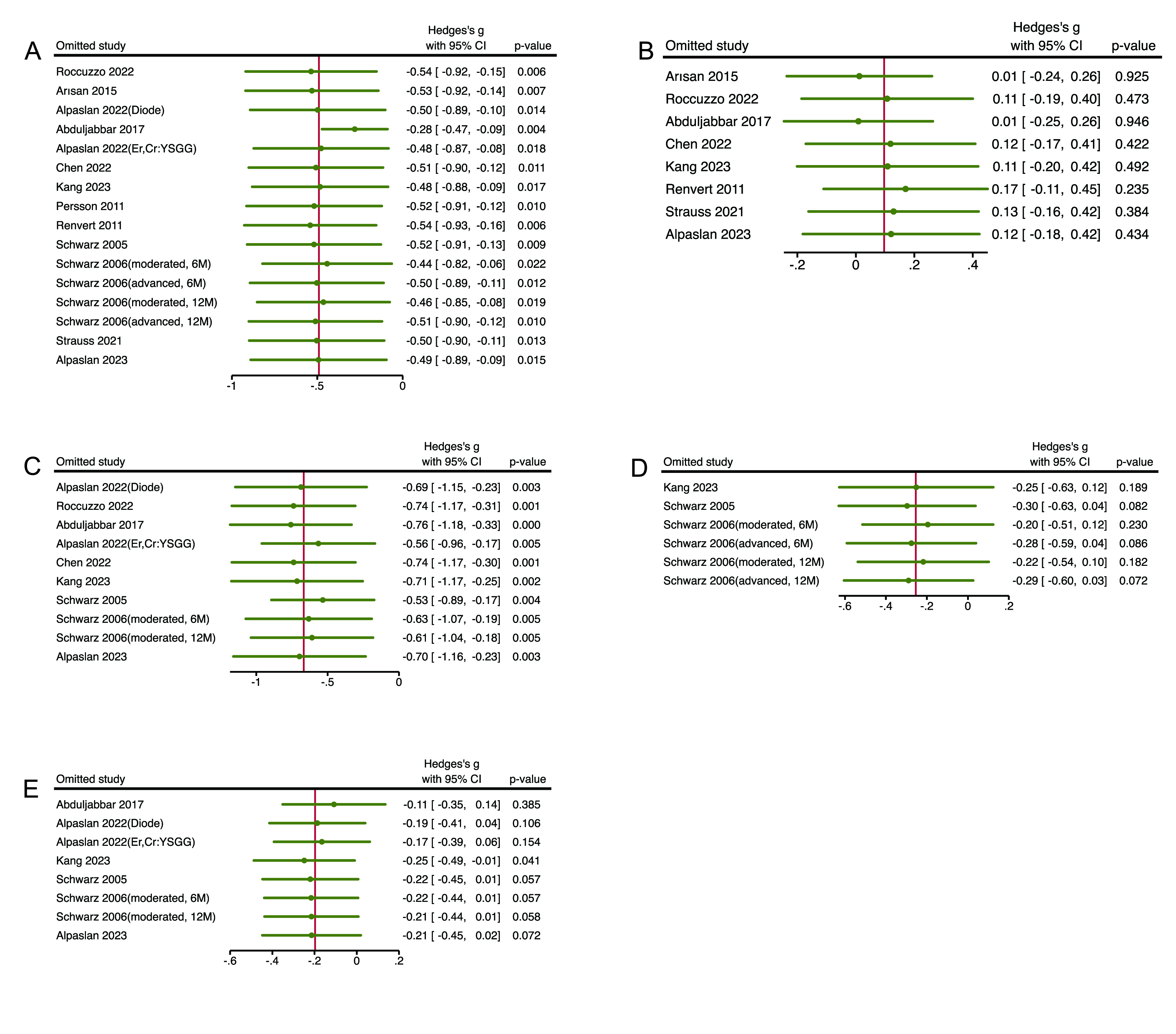

Supplement: Supplementary file 5 — Supplementary Material 5: Fig. S3: Sensitivity analysis of primary and secondary outcomes. (A) PD; (B) bone loss; (C) BOP; (D) CAL; (E) PI. [file 40729_2024_570_MOESM5_ESM.tif]
